# Dual Network Hydrogels Incorporated with Bone Morphogenic Protein-7-Loaded Hyaluronic Acid Complex Nanoparticles for Inducing Chondrogenic Differentiation of Synovium-Derived Mesenchymal Stem Cells

**DOI:** 10.3390/pharmaceutics12070613

**Published:** 2020-06-30

**Authors:** Qing Min, Jiaoyan Liu, Yuchen Zhang, Bin Yang, Ying Wan, Jiliang Wu

**Affiliations:** 1 School of Pharmacy, Hubei University of Science and Technology, Xianning 437100, China; baimin0628@hbust.edu.cn (Q.M.); zhangych@hbust.edu.cn (Y.Z.); 2 College of Life Science and Technology, Huazhong University of Science and Technology, Wuhan 430074, China; liujiaoyan@hust.edu.cn (J.L.); m201871739@hust.edu.cn (B.Y.)

**Keywords:** dual network hydrogel, complex nanoparticles, bone morphogenic protein-7, chondrogenic differentiation, synovium-derived mesenchymal stem cells

## Abstract

Alginate-poloxamer (ALG-POL) copolymer with optimal POL content was synthesized, and it was combined with silk fibroin (SF) for building ALG-POL/SF dual network hydrogels. Hyaluronic acid(HA)/chitosan-poly(dioxanone)(CH-PDO) complex nanoparticles (NPs) with optimized composition and high encapsulation efficiency were employed as a vehicle for loading bone morphogenic protein-7 (BMP-7). BMP-7-loaded HA/CH-PDO NPs were incorporated into ALG-POL/SF hydrogel for constructing composite gels to achieve controlled release of BMP-7. These gels showed thermosensitive sol-gel transitions near physiological temperature and pH; and they were tested to be elastic, tough and strong. Some gels exhibited abilities to administer the BMP-7 release in nearly linear manners for a few weeks. Synovium-derived mesenchymal stem cells (SMSCs) were seeded into optimally fabricated gels for assessing their chondrogenic differentiation potency. Real-time PCR analyses showed that the blank ALG-POL/SF gels were not able to induce the chondrogenic differentiation of SMSCs, whereas SMSCs were detected to significantly express cartilage-related genes once they were seeded in the BMP-7-loaded ALG-POL/SF gel for two weeks. The synthesis of cartilaginous matrix components further confirmed that SMSCs seeded in the BMP-7-loaded ALG-POL/SF gel differentiated toward chondrogenesis. Results suggest that BMP-7-loaded ALG-POL/SF composite gels can function as a promising biomaterial for cartilage tissue engineering applications.

## 1. Introduction

Autologous chondrocyte implantation is one of clinically usable techniques for treating articular cartilage injuries, which is usually implemented by filling cartilage defect site with high numbers of autologous chondrocytes by the aid of certain supporting materials [1,2]. Taking into account the fact that the population of chondrocytes in articular cartilage is very small, the chondrocytes harvested from non-load-bearing areas must be expanded in vitro to a sufficient number. However, two-dimensional culture-expansion of chondrocytes could cause their dedifferentiation to some degree, depending on the passage of cells, and in turn, result in a loss of capacity to form stable hyaline cartilage in vivo [3,4,5]. This limitation has evoked a great deal of interest in using stem cells as a cell source for cartilage repair. Several types of stem cells, including bone marrow-derived mesenchymal stem cells (BMSCs), adipose-derived stem cells (ASCs) and synovium-derived mesenchymal stem cells (SMSCs), have been investigated for the usage in cartilage repair [6]. Despite their applicability, some evidence supports that SMSCs can retain high transplant survival rates and undergo rapid proliferation and chondrogenic differentiation when compared to BMSCs and ASCs [7,8,9,10].

Polymer carriers in the form of scaffolds, microspheres and hydrogels have been commonly used in tissue engineering. Among them, hydrogels attract a lot of attention as they can be utilized in a minimally invasive manner, and are able to form into solid-like objects in situ with arbitrary shapes that are closely aligned with the morphology of irregular defects [11]. In addition, polymer hydrogels have network structure featuring with high water content, compliant elasticity, and facile diffusion of biomolecules, making them attractive for building three-dimensional (3D) extracellular matrix (ECM) mimics. Furthermore, the sol-gel transition nature of hydrogels allows them for conveniently carrying cells while effectively controlling cell density and distribution [12].

Hydrogels based on certain natural polymers are advantageous and preferable for biomedical applications because of their good biocompatibility and biodegradation. Among them, Ca^2+^ ion-crosslinked alginate hydrogels have been largely investigated for various applications. Nevertheless, such crosslinked alginate hydrogels often show poor stability and uncontrollable properties owing to the exchange between Ca^2+^ ions and monovalent cations (such as Na^+^ and K^+^) hailing from the host tissue [13]. Another type of alginate hydrogel with thermosensitive characteristics was developed by using alginate-poloxamer (ALG-POL) copolymers [14], but these gels appeared to be mechanically weak and brittle in nature due to their high percentage of easily collapsed Pluronic F127 component [14,15].

Natural polymer hydrogels with a single network often show low mechanical strength, rendering them less suitable for certain applications where sufficient strength and sustained dimensional stability are concurrently required. Many studies have revealed that the polymer hydrogel with dual or multiple networks could have large enhancement in their dimensional stability, mechanical performance and degradation tolerance when compared to the single network gel [16,17,18]. Therefore, it is rational to combine ALG-POL with other natural polymers to build dual network hydrogels for tackling the weak and brittle problems related to ALG-POL gels themselves. Silk fibroin (SF), a kind of fibrous protein, can be processed into hydrogels through enzyme-catalyzed crosslinking [19], but the applied amount of H_2_O_2_, one of co-agents for crosslinking SF, must be controlled lower than a certain threshold to ensure the safe use of resulting SF gels in vivo. In this study, an attempt was made to build a type of dual network ALG-POL/SF hydrogel with desirable composition and properties for delivering SMSCs in view of the chondrogenic virtues of SMSCs. SMSCs have been shown to have great potential to differentiate into functional cells, such as osteoblasts, chondrocytes, adipocytes and neural cells [6]. Hence, the employed ALG-POL/SF hydrogel should provide chemical or biological cues to guide chondrogenic differentiation of SMSCs, besides serving as a support for delivering and housing SMSCs. In this circumstance, combined delivery of SMSCs with certain chondrogenic factors may be a feasible strategy for inducing the chondrogenic differentiation of SMSCs. Different kinds of growth factors, such as bone morphogenetic proteins (BMPs), transforming growth factors and insulin-like growth factors, have been explored for potential in promoting chondrogenic differentiation of mesenchymal stem cells [20,21]. In the BMP factor family, BMP-7 is known to act as a critical cytokine that induces chondrogenic differentiation of mesenchymal stem cells and has pro-anabolic and anti-catabolic effects on cartilage tissue restoration [22,23]. In this study, BMP-7 was therefore delivered together with SMSCs using ALG-POL/SF gel as a vehicle in order to actualize the chondrogenic differentiation of SMSCs. Generally, direct incorporation of growth factors within a hydrogel would result in initial burst release due to the porous structure and high water content of the gel, which is quite disadvantageous to factor utilization [24]. Studies revealed that BMP-7 had highly sensitive concentration dependence in chondrogenesis modulation, and its dose needed to be well controlled within a proper range for effectively stimulating chondrogenesis, and consequently inducing the formation of articular hyaline cartilage in vivo [23,25]. Accordingly, the incorporated amount of BMP-7 and its release have to be finely regulated in accordance with the early chondrogenic stage. In our previous study, a type of hyaluronic acid(HA)/chitosan-poly(dioxanone)(CH-PDO) complex nanoparticles (NPs) was constructed and served as a carrier for delivering BMP-2 [26]. The optimal HA/CH-PDO NPs demonstrated the capacity for high BMP-2 loading efficiency, controlled BMP-2 release kinetics and bioactivity preservation of the released BMP-2. Under the present circumstances, HA/CH-PDO NPs were used to carry BMP-7 in order to achieve sustained and dose-adjustable BMP-7 release.

The thrust of this study is to build a type of ALG-POL/SF hydrogel embedded with BMP-7-loaded HA/CH-PDO NPs for delivering SMSCs while effectively inducing their chondrogenic differentiation. Results related to preparation and characterization of gels, BMP-7 release and differentiation analysis of SMSCs were reported.

## 2. Materials and Methods

### 2.1. Materials

Sodium alginate (ALG, M_n_: 1.3 × 10^5^), Poloxamer 407 (POL, M_n_: 12,600), 1-ethyl-3- (3-dimethylaminopropyl)-carbodiimide (EDC), horseradish peroxidase (HRP), *N*-hydroxyl succinimide (NHS), and chitosan (CH, M_n_: 1.2 × 10^5^; deacetylation degree: ca.92%) were procured from Aladdin (Shanghai, China). Hyaluronic acid (HA, sodium salt, M_w_: 90–110 kDa), and *p*-dioxanone were purchased from Sigma-Aldrich (Shanghai, China). Recombinant human BMP-7 and BMP-7 ELISA kit were purchased from R&D systems (Minneapolis, MN, USA). Other reagents and chemicals were of analytical grade and purchased from Sinopharm (Shanghai, China).

SF was produced using Bombyx Mori cocoons according to the reported method [17,27]. A 10% SF solution was prepared and stored at 4 °C for the subsequent use. Details for the preparation of SF solution can be found in the supplementary data file.

Alginate-poloxamer (ALG-POL) copolymers were synthesized via a two-step method. POL was first modified to produce an intermediate, monoamine-terminated POL (MATP), according to reported methods [28,29], and then, the obtained MATP was coupled onto ALG backbone to prepare ALG-POL copolymers. An ALG-POL copolymer with POL percentage of around 66 wt% was used for gel preparation. Details for synthesizing MATP and ALG-POL copolymers, and the Fourier transform infrared (FTIR) spectra (Figure S1) as well as relevant analysis for ALG-POL are also provided in the supplementary data file.

### 2.2. Preparation of Nanoparticles

CH-PDO with a PDO percentage of about 30 wt% was synthesized using the method described in our previous study [26]. Blank HA/CH-PDO NPs were first prepared. CH-PDO was dissolved in 1.0% acetic acid to prepare a 0.6 mg/mL solution, and HA was dissolved in deionized water to produce a 1.2 mg/mL solution, respectively. 150 μL of sodium tripolyphosphate (TPP) solution (0.75 mg/mL) was introduced into 1 mL of HA solution and the resulting HA/TPP solution was then mixed with the CH-PDO solution at a HA/CH-PDO mass ratio of 4:3. After that, the mixture was stirred for 20 min for the complete stabilization of NPs, and the resulting NPs were collected by centrifugation. The obtained NPs were lyophilized for further use.

The similar method was used to prepare BMP-7-encapsulated HA/CH-PDO NPs. A given amount of BMP-7 was introduced into a stored CH-PDO solution with gentle stirring, and the resulting solution was mixed with the prepared HA/TPP solution at the same HA/CH-PDO mass ratio of 4:3, followed by the same processing described above for producing BMP-7-encapsulated HA/CH-PDO NPs.

BMP-7 content in HA/CH-PDO NPs was determined using a BMP-7 ELISA Kit, and encapsulation efficiency (EE) of NPs was calculated by the following equation:

EE(%) = (*M*_0_*/M*_1_) × 100% (1)

where *M*_0_ is the mass of BMP-7 loaded inside HA/CH-PDO NPs, and *M*_1_ is the feed mass of BMP-7.

### 2.3. Preparation of Hydrogels

BMP-7-free composite solutions were prepared using ALG-POL, SF solution, blank HA/CH-PDO NPs, HRP and H_2_O_2_, and these composite solutions were used to build blank gels for composition and property evaluation in order to save costly BMP-7. In a typical procedure, one of composite solutions was introduced into a vial and it was stirred in an ice/water bath for 5 min. The vial was then incubated in a water bath at 37 °C until the solution became a gel. During the incubation, the fluidity of the solution was examined by regularly inverting the vial, and gelation time was recorded starting from the vial incubation in the 37 °C water bath and ending at the moment when the solution stopped flowing. The composite solutions containing BMP-7 were also prepared by adding the BMP-7-loaded HA/CH-PDO NPs into the aqueous ALG-POL/SF mixture, and the resulting mixtures were further processed into BMP-7-loaded ALG-POL/SF gels using the same method applied to the blank gels. The major parameters for these solutions and resulting gels are summarized in Table 1 and Table 2, respectively.

### 2.4. Characterization

The morphology of HA/CH-PDO NPs with or without BMP-7 load was viewed with a transmission electron microscope (TEM, Tecnai G2, FEI, Hillsboro, OR, USA). Their hydrodynamic size and zeta (ζ) potential were measured using a dynamic light scattering (DLS) instrument (Nano-ZS90, Malvern Instruments, Worcestershire, UK). Rheological measurements of blank composite solutions were performed on a rheometer (Kinexus Pro KNX2100, Southborough, MA, USA) that was equipped with a stainless steel parallel-plate sample holder. Temperature sweep curves were recorded in the range of 25 to 45 °C by heating the solutions at a temperature-elevated rate of 1 °C/min, and their incipient gelling temperature (T_i_) was determined at the intersection point of storage modulus (G′) and loss modulus (G′′). Shear-dependence viscosity of composite solutions was measured at 25 °C in a shear rate range between 0.1/s and 200/s. The isothermal frequency dependence of G′ and G′′ was measured in a frequency range between 0.1 and 100 Hz at 37 °C and a constant strain of 1%. The strain sweep spectra of G′ and G′′ were also detected at 37 °C and 1 Hz and used to estimate the elasticity of some blank gels.

### 2.5. Mechanical Tests

Unconfined compression tests of blank gels were carried out using a mechanical testing machine (MACH-1™, Biomomentum, Laval, Canada). Cylindrical samples (10 mm diameter and 7–8 mm height) were compressed at a constant strain rate of 10%/min. The precise height of samples was determined by applying a preload threshold (5 mN) to establish contact between the sample and the upper platen before initiation of the compression tests.

Hysteresis testing of blank gels was performed by subjecting the gel samples to 10 discontinuous loading–unloading cycles employing a constant strain rate of 10%/min while compressing to a strain of 50%. At the ending point of each cyclic test, the strain was kept at zero position for 10 min to allow the restoration of 3D shape of samples, and the next step was then applied and so forth. All tests were carried out in a sample bath of PBS to diminish the effects of water content.

Compressive modulus (E) of blank gels was determined using the slope of a linear fit to compressive stress-strain curve (loading portion of hysteresis loop) over 2–5% strain.

Strain-recovery rate (R%) is defined as

R(%) = [*h*(*t*)/*h*_0_] × 100% (2)

where *h*_0_ is the original height of the gel sample, and *h*(*t*) is the recovered height of the gel sample after unloading.

The dissipation energy (ΔU), also called as hysteresis energy, of gel samples is defined as the area of the hysteresis loop encompassed by the loading-unloading curve

(3) $$\Delta U=\int_{loading}\sigma_{comp}d\varepsilon -\int_{unloading}\sigma_{comp}d\varepsilon$$

### 2.6. Release of BMP-7

Cylindrical gel samples embedded with BMP-7-loaded HA/CH-PDO NPs were tested to determine BMP-7 release profiles. Each (0.5 mL) of composite solutions (see Table 2 for their composition) was filled into a cylindrical mold (diameter: 10 mm) and incubated at 37 °C for 20 min for gel formation. The resulting gel samples were individually immersed in 3 mL of PBS in vial with shaking at 60 rpm and 37 °C. At predetermined time intervals, 0.5 mL of release medium was withdrawn and the same volume of fresh buffer was replenished. The cumulative amount of the released BMP-7 was measured using a BMP-7 ELISA Kit according to the manufacturer’s instructions. Release profiles for BMP-7-loaded HA/CH-PDO NPs and for ALG-POL/SF gels directly loading with BMP-7 were also detected in a similar way and used for making comparisons.

### 2.7. Isolation and Phenotypic Analysis of SMSCs

Sprague Dawley rats (12 weeks old) were used as cell donors. Isolation and culture of SMSCs were performed following reported methods [9,30]. Synovial tissues were aseptically excised from the knee joints of rats. All animal experiments were approved by the Animal Care and Use Committee of Hubei University of Science and Technology (2019S669). The synoviums were finely minced into tiny pieces under a dissecting microscope using scalpels and digested in a collagenase type II solution (0.2%) for 3 h at 37 °C. The digested pieces were filtered through a 40 μm nylon strainer to yield a single-cell suspension and cells were obtained by centrifuging at 1500 rpm for 5 min. Nucleated cells isolated from synovium were then seeded in culture dishes (90 mm diameter) at 1 × 10^4^ cells/60-cm^2^ for 7 days as Passage 0. Dishes were trypsinized and cells were harvested to count cell colony number. Cells were subcultured 14 days as Passage 1. All these cells were cultured in complete medium consisting of α-minimal essential medium (α-MEM) containing 20% fetal bovine serum (FBS), 100 U/mL penicillin, 100 μg/mL streptomycin, and 2 mM l-glutamine and incubated at 37 °C with 5% CO_2_.

Colony-forming assay was conducted according to the method described in the literature [9,30]. 100 cells at Passage 1 were plated in 90 mm dishes and cultured in complete medium for 7 days. Subsequently, the cells were fixed with 4% paraformaldehyde, stained with 0.5% crystal violet in 4% paraformaldehyde for 5 min, and washed twice with distilled water. The colony number was counted and those colonies having their diameter less than 2 mm and being faintly stained colonies were ignored.

The surface epitope profiles of SMSCs were analyzed via flow cytometry (FCM). The following mouse anti-rat monoclonal fluorescein isothiocyanate (FITC)-coupled antibodies (BioLegend, San Diego, CA, USA): CD11b, CD45 and CD90 were used for testing. FITC-coupled nonspecific mouse IgG antibodies (BioLegend, USA) were used as isotype control. Briefly, cells (1 × 10^5^, Passage 3) were suspended in 500 μL PBS containing the mentioned antibodies (20 ng/mL). After incubation for 30 min at 4 °C, the cells were washed with PBS and resuspended in 1 mL PBS for analysis using a flow cytometer (BD FACS Calibur, San Jose, CA, USA) together with CellQuest software (version 5.1).

### 2.8. Cell Proliferation

ALG-POL solutions and SF solutions were respectively introduced into glass dishes to form thin-layer, followed by sterilization under UV light at 4 °C for 4 h. Two types of solutions were blended at prescribed ratios while being added with HRP and H_2_O_2_ to produce three kinds of ALG-POL/SF solutions with their proportions as the same as that assigned for GB-1, GB-2 and GB-3 gels (see Table 1). For cell seeding, an aliquot of each ALG-POL/SF solution was homogeneously mixed with a given volume of α-MEM containing SMSCs to produce mixtures with a cell density of 5 × 10^6^ cells/mL. The cell-containing mixtures were plated onto 24-well culture dishes (200 μL/well) and incubated at 37 °C for gel formation. The gels were then cultured in α-MEM (20% FBS) for varied durations up to 7 days with medium replacement every 2 days. At the end of preset time points, gels were washed with PBS, frozen in liquid nitrogen, and crushed into powder. Each powder sample was digested in proteinase K solution at 55 °C for 48 h. The supernatant for each sample was collected. The DNA content in these supernatants was determined using a Quant-iT PicoGreen dsDNA Kit (Invitrogen) while following the manufacturer’s instructions (λ_ex_: 480 nm, λ_em_: 528 nm). Two-dimensional culture of SMSCs in complete medium was used as control.

### 2.9. Real-Time Polymerase Chain Reaction (RT-PCR) Analysis

SF, ALG-POL and BMP-7 loaded HA/CH-POD NPs were sterilized with ethylene oxide, and prepared into two kinds of solutions together with HRP and H_2_O_2_ with the composition the same as that shown in Table 2. A given volume of culture medium containing Passage 3 SMSCs was respectively mixed with two kinds of the prepared solutions to produce cell-incorporated mixtures with a cell density of 10^7^ cells/mL. To each well of 24-well culture plates, an aliquot (200 μL) of the mixtures was pipetted, and plates were cultured at 37 °C for gel formation. The gels were further cultured with maintenance medium (2% FBS) in a humidified incubator (37 °C and 5% CO_2_) for 14 days, and the medium was refreshed every 2 days. SMSCs were also seeded in GB-1 gel without BMP-7 loading (see Table 1) at the same cell density, and the resulting gel was also cultured in maintenance medium for 14 days and used for comparison. SMSCs cultured under monolayer condition in maintenance medium were used as control.

After incubation, the cell-loaded gels were frozen in liquid nitrogen, and crushed into powder. To each sample, 1 mL of TRIzol reagent (Invitrogen, Shanghai, China) was added to isolate RNA according to manufacturer’s instructions. The isolated RNA was quantified using a Quant-iT RNA Assay Kit (Invitrogen). RNA (1 μg) was then reverse transcribed into complementary DNA (cDNA) using a High-Capacity cDNA Reverse Transcription Kit (Applied Biosystems, Shanghai, China) following manufacturer’s protocol. The obtained cDNA was subjected to PCR to examine the gene expression of SOX-9, aggrecan and collagen type II (COL II) using a commercially available SYBR GreenER qPCR Supermix Kit (Invitrogen). Relative gene expression levels were calculated according to the 2^−ΔΔCt^ method with normalization to glyceraldehyde-3-phosphate dehydrogenase (GAPDH). The employed primer sequences are listed in Table S1.

### 2.10. Analysis of Matrix Components

GEL-2 gel seeded with SMSCs was used to assess the deposition of two cartilage-specific matrix components, COL II and glycosaminoglycan (GAG), taking account of the higher initial BMP-7 load in GEL-2 gel (Table 2). After being cultured for various periods up to 21 days, the cell-seeded gels were fixed in a 2.5% glutaraldehyde solution for 4 h at 4 °C and dehydrated with a graded ethanol series to 100% ethanol, followed by air-drying. The dry gel samples were then embedded in paraffin, and sectioned into slices with a thickness of 6 μm using a microtome (Leica EG 1160, Leica Microsystems, Nussloch, Germany). The resulting slices were stained with toluidine blue for detecting GAG deposition. COL II deposition was detected using immunostaining. Briefly, the deparaffinized and rehydrated slices were incubated with 0.1% Triton X-100 in PBS for 15 min and blocked with 5% bovine serum albumin in PBS for 30 min. They were then incubated with the primary antibody (mouse anti-rat monoclonal COL II antibody, Abcam, Cambridge, MA, USA), followed by incubation with Alexa Fluor 488 labeled goat anti-mouse secondary antibody (Abcam, USA) after washing with PBS. The stained samples were imaged using a fluorescence microscope (Leica DMI6000B, Germany).

At prescribed time intervals, the cell-seeded gels were treated with the same method as that used for DNA isolation to obtain supernatants for different samples. GAG content in the supernatants was determined via a 1,9-dimethylmethylene blue (DMMB) binding assay [17]. Chondroitin sulfate was used to generate a standard curve. COL II amount in the supernatants was measured using a COL II BioAssay ELISA kit (US Biological, Swampscott, MA, USA) in accordance with the manufacturer′s protocol.

### 2.11. Statistical Analysis

Data were presented as mean ± standard deviation. Analysis of the difference (SPSS 20 for windows) between groups was performed using one-way ANOVA. Statistical difference was declared at *p* < 0.05.

## 3. Results and Discussion

### 3.1. Complex Nanoparticles and Their Gel Construction

**Figure 1:**
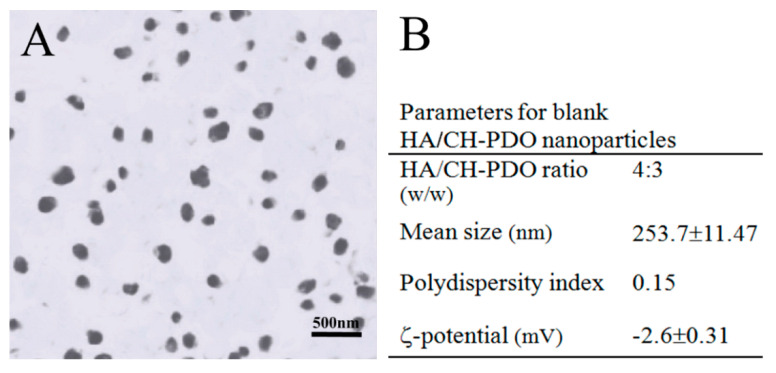

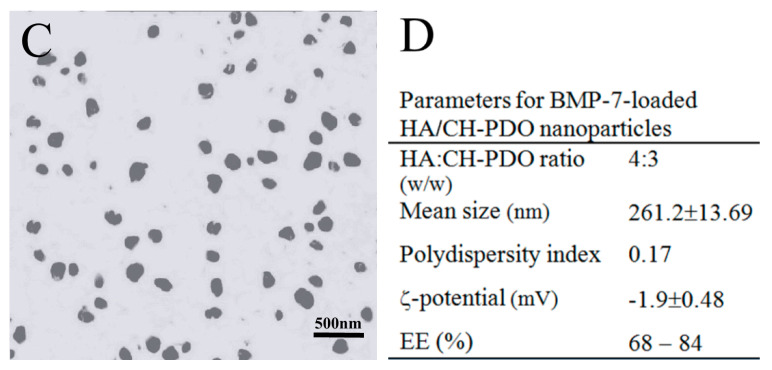
Representative TEM micrographs and major parameters for blank HA/CH-PDO NPs (**A**,**B**) and BMP-7-loaded HA/CH-PDO nanoparticles (NPs) (**C**,**D**).

Blank HA/CH-PDO NPs were first prepared to optimize their composition and major parameters. Figure 1A,B show a representative TEM image and major parameters for blank HA/CH-PDO NPs. These NPs were seen to be approximately spherical with various sizes changing from several tens of nanometers to around 300 nm, and they had small negative ζ-potential of around −2 mV. Incorporation of BMP-7 into HA/CH-PDO NPs did not impose significant effects on the morphology, mean size and ζ-potential of the resulting NPs (Figure 1C,D). It is worth pointing out that the composition for BMP-7-loaded HA/CH-PDO NPs was formulated as a HA/CH-PDO ratio of 4:3 in this study in order to achieve high EE while enabling the surface charge of the resulting NPs to be nearly neutral (Figure 1D).

CH is a cationic polysaccharide and it has been extensively investigated for diverse biomedical applications due to its many advantages such as biocompatibility, biodegradability, anti-microbial activity, bioadherence and cell affinity [31,32]. Despite their wide applicability, CH microspheres or NPs generally are incompetent for administering long-term release of drugs or biomolecules because of their burst release features even though they are crosslinked with varied kinds of crosslinkers [26,32,33]. Poly(dioxanone) (PDO) is a kind of biodegradable and biocompatible polyester with mechanically strong and tough properties [34]. In our previous study, PDO oligomers were grafted onto the C-6 sites of CH to produce a type of CH-PDO copolymer with soluble features in aqueous media whilst having free amino groups at the C-2 sites of CH backbone [26]. Like CH, such a synthesized CH-PDO copolymer still has its cationic characteristics and can be used together with other anionic polysaccharides for preparing complexes. HA is an anionic polysaccharide composed of *N*-acetyl-d glucosamine and d-glucuronic acid units and it exists in many types of ECM in the human body as an important component for facilitating cell locomotion, proliferation and phenotype preservation [35,36]. By combining HA with CH-PDO, the constructed HA/CH-PDO complex NPs demonstrated their promising potential in delivering bioactive molecules because they were built via ionic reactions between amino groups in CH backbone and carboxyl groups in HA without involving any covalent crosslinking or harsh processing environment. As a result, HA/CH-PDO complex NPs were able to preserve the bioactivity of the loaded protein factor [26]. As mentioned earlier, the BMP-7-loaded HA/CH-PDO NPs will be used to embed into thermosensitive ALG-POL/SF hydrogel for building composite gels. The primary investigations revealed that the surface charge of HA/CH-PDO NPs could influence the sol-gel transition temperature of the resulting composite gels to some extent. Another concern for preparation of NPs is that these HA/CH-PDO NPs need to have high EE for the rational use of BMP-7 due to the high cost of BMP-7. Accordingly, HA/CH-PDO NPs were designed at such compositional ratio (Figure 1D) in order to endow the BMP-7-loaded HA/CH-PDO NPs with near-neutral charge surface while having high EE, allowing them to be suitable for the subsequent construction of composite gels.

**Figure 2:**
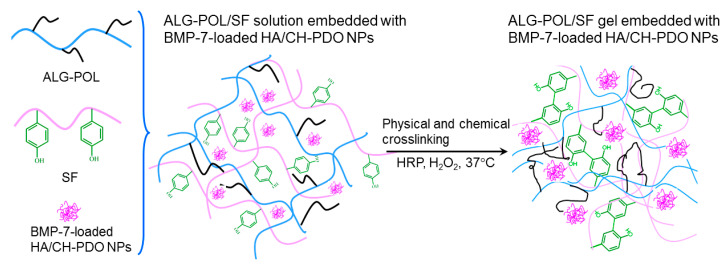
A schematic representation for formation of alginate-poloxamer (ALG-POL)/silk fibroin (SF) composite gel.

ALG-POL is a type of thermoresponsive copolymer and their solutions can transform into gels under temperature stimulation, which results in formation of physically crosslinked network inside the resulting gels [14,15]. The tyrosine residues in SF molecules can be chemically crosslinked via the mediation of co-agents, namely, HRP and H_2_O_2_ [27,37]. Therefore, it is feasible to build a new type of ALG-POL/SF hydrogel with physically and chemically crosslinked networks on account of their independent gelable mechanisms. Figure 2 shows a schematic representation for the construction of ALG-POL/SF composite gel. The resulting gel is intended for use in delivering both BMP-7 and cells.

### 3.2. Properties of Gels Embedded with Blank HA/CH-PDO NPs

A previous study stated that ALG-POL alone was gelable when its solution concentration reached about 15 wt% or higher [14]. In the current situation, the synthesized ALG-POL with optimal POL content can be processed into gels when its solution concentration reached 12 wt% or higher. Nevertheless, ALG-POL gels were found to be weak and brittle. Figure S2 elucidates strain sweep curves of G′ and G′′ for several ALG-POL gels. All these ALG-POL gels were seen to have their yielding strains at around 8% or less, revealing that they are inelastic. In addition, it is observed that the G′ values of ALG-POL gels corresponding to three concentrations of 12, 14 and 16 wt% in their respective linear viscoelastic regions (LVR) are around 700, 900 and 1200 Pa, respectively, implying that they are mechanically weak [17].

In the present study, it was found that an ALG-POL solution was not gelable even being incubated at 37 °C for a long period of time if its concentration was lower than 10 wt%. Nevertheless, the ALP-POL in ALG-POL/SF gels can function as an effective component to endow ALG-POL/SF gels with thermoresponsive features even though its concentration in ALG-POL/SF gels was set as 5 wt% (Table 1).

**Figure 3:**
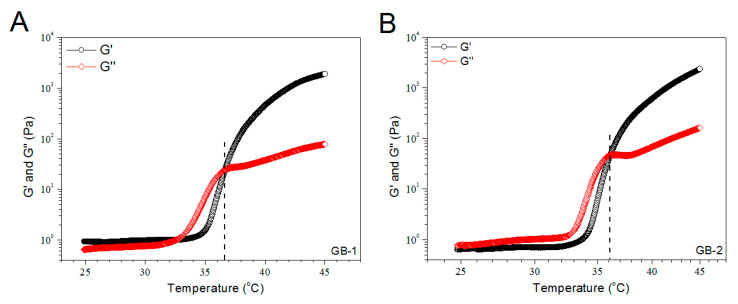

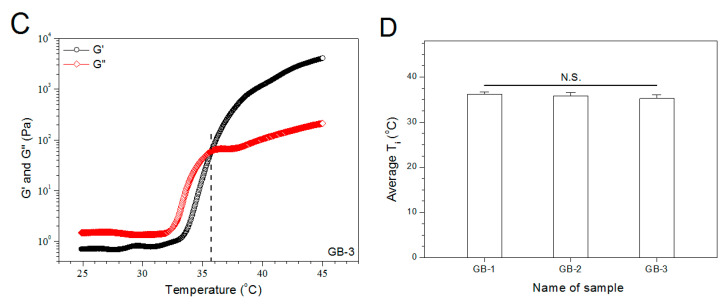
Temperature dependencies (**A**–**C**) of G′ and G′′ and incipient gelling temperature (**D**) for composite gels without bone morphogenetic protein (BMP)-7 load.

Figure 3 exhibits the representative temperature dependences of G′ and G′′ for the gels illustrated in Table 1. The gels showed their T_i_ at around 36 °C. More gel samples were detected and their average T_i_ values are depicted in Figure 3D. The bar-graph points out that incorporation of blank HA/CH-PDO NPs into ALG-POL/SF gel did not significantly alter T_i_ of ALG-POL/SF gel itself. Additionally, data in Table 1 show that pH and gelation time of these gels were similar to each other, indicative of their applicability under physiological conditions.

These blank gels were detected to determine their frequency sweep of G′ and G′′ for estimating their strength, and results are represented in Figure S3. The G′ values of ALG-POL/SF gels in their LVR of frequency sweep was much larger than that for ALG-POL gels (see Figure S2) and measurably increased as the incorporated amount of HA/CH-PDO NPs changed from 0 to 2 wt%. These curves suggest that significant enhancement in strength can be achieved by combining ALG-POL and SF together when compared to singular ALG-POL component gels, and incorporation of HA/CH-PDO NPs into ALG-POL/SF gels can further improve their strength.

Taking account of the injectable applicability of these gels, they were tested for their viscosity versus shear rate, and results are elucidated Figure S4. Three kinds of gels were viscous over the shear-rate sweep range but their viscosity dropped as the applied shear rate increased, showing explicit shear-thinning features. Considering that the gel injection is usually conducted at room temperature, results in Figure S4 demonstrate that these gels have well-defined injectability.

**Figure 4:**
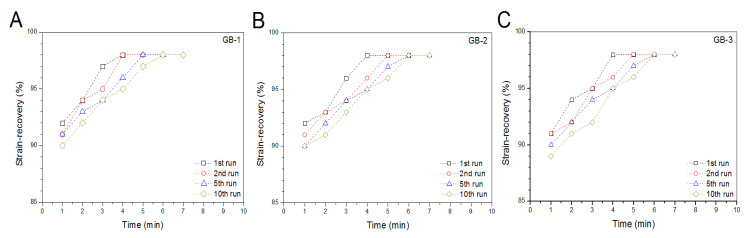
Strain–recovery of gel samples with compression deformation of 50% related to their original height during four discontinuous loading-unloading cycles.

**Figure 5:**
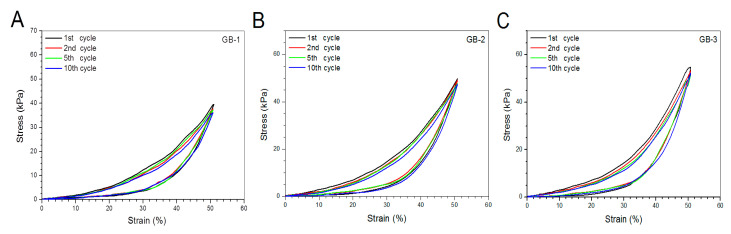
Stress–strain hysteresis plots of gel samples during the 1st, 2nd, 5th and 10th cycles of loading and unloading to a strain magnitude of 50%.

**Figure 6:**
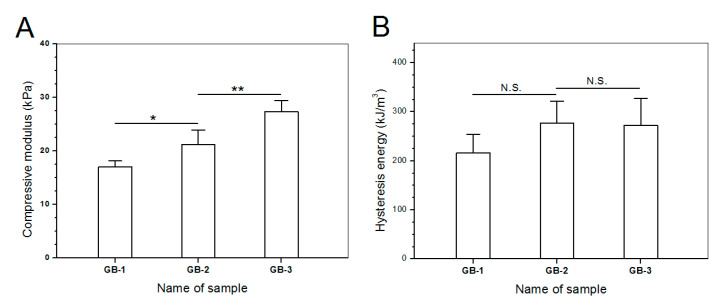
Compression modulus (**A**) and dissipation energy changes (**B**) for different gel samples (*, *p* < 0.05; **, *p* < 0.01; N.S., no significance).

Blank gels were subjected to compression measurements by compressing them to 50% strain in a loading-unloading manner to examine their strain recovery, and relevant data are graphed in Figure 4. These blank gels were able to quickly restore to around 90% of their respective original heights within 1 min, and their strain recovery reached to 98% in 6 min after being discontinuously compressed for 10 times, demonstrating their high elasticity. Cyclic loading-unloading compression tests were performed to examine the toughness of these blank gels, and results are shown in Figure 5. Curves in Figure 5A elucidate that the loading stress for GB-1 gel slowly increased until the strain reached about 10%, and it continued to faster rise to around 40 kPa when the strain was extended to 50%; during the dimensional recovery of GB-1 gel in the unloading procedure, a hysteresis loop was formed in the first cycle. The hysteresis loops for the following three loading-unloading cycles were nearly overlapped. In comparison to GB-1 gel, GB-2 and GB-3 gels also had approximately overlapped hysteresis loops in their respective loading-unloading curves during their cyclic measurements. The hysteresis loops for GB-2 gel looked steeper with a significantly higher stress at the ending-point of 50% strain when compared to that for GB-1 gel. The same thing was true when comparisons were made between GB-2 and GB-3 gels. Compression modulus and hysteresis energy for these blank gels were calculated and relevant data are provided in Figure 6. It can be observed that there were significant differences in modulus among these gels. The modulus of ALG-POL/SF gel was tested to be markedly enhanced by incorporating HA/CH-PDO NPs, and also, was significantly dependent on the incorporated amount of HA/CH-PDO NPs. In contrast to these observations shown in Figure 6A, the blank gels had similar hysteresis energy without significant difference, signifying their similar toughness (Figure 6B). Data numerated in Table 1 denote that these blank gels had the same matrix with small differences in the incorporated amount blank HA/CH-PDO NPs. As described in the experimental section, the blank HA/CH-PDO NPs were physically blended with other components to construct GB-2 and GB-3 gels, and hence, they would fill some pores inside these gels when compared to GB-1 gel without incorporation of any NPs. As a result, the incorporated HA/CH-PDO NPs actually function to increase the density of GB-2 and GB-3 gels, and in turn, enhance their strength. Since these blank HA/CH-PDO NPs were randomly distributed throughout gel matrix without any chemical linkages to the components of the gels, and accordingly, they would impose a limited impact on the deformation of network formed by molecular chains, leading to insignificant effect on the hysteresis energy of the gels because hysteresis energy is closely correlated to the capability of the gel to resist the network deformation [12,18].

Cartilage is a type of specialized soft tissue that covers the surface of diarthroidal joints. It fulfills essential mechanical functions such as load bearing and shock absorption while providing a smooth surface for nearly frictionless joint movements [38]. Nowadays, it has been recognized that hydrogels used for cartilage repair need to be strong, elastic and tough because the applied gels have to function as a support like cartilage ECM for housing the seeded cells or the cells migrated from the host tissue while having sufficient capacity to resist the repeated dynamic shocks without collapsing or crushing [11,39]. Accordingly, the newly developed composite gels in this study must be strong enough with reliable elasticity and toughness.

SF is a type of fibrous protein and has robust mechanical properties in wet state with in vivo degradation tolerance owing to its specific structure featured with chain conformation transformation from α-helix to β-sheet under certain conditions [19]. SF hydrogels could be strong and elastic, relying on the employed crosslinkers and the crosslinking degree [27,37]. Nevertheless, SF hydrogels crosslinked with safe doses of H_2_O_2_ and HRP for in vivo use are commonly weak, indicated by their low elastic modulus (ca.1 kPa) and viscous modulus (ca.10 Pa) [17]. In the present study, primarily mechanical measurements in intermittent mode were conducted to ascertain the feasibility for constructing mechanically strong ALG-POL/SF gels as well as their composites. It is known that hydrogels are a class of soft objects with deformable characteristics and varied properties, depending on their composition and structure. There is no a general criterion currently available for confining the strain range of hydrogels when they are subjected to compression measurements [18,38,40]. In this instance, the strain for the unconfined compression test of blank composite gels was selected as 50%, as described in the experimental section. Such selection was made considering that the implanted gel for the repair of cartilage defects with full thickness usually faces a confined mechanical environment and the thickness deformation of the applied gels will be much less than 50% [41]. Curves in Figure 4, Figure 5 and Figure 6 exhibit that ALG-POL/SF gel and their composites are able to almost fully self-recover to their original dimension within a short period of time, and they also have high modulus with large energy dissipation higher than 200 kJ/m^3^. These results demonstrate that the presently devised dual network gels are strong, elastic and tough, and they potentially meet the mechanical requirements in cartilage repair.

### 3.3. BMP-7 Release from NPs and Gels

On the basis of above investigations, BMP-7-loaded HA/CH-PDO NPs were embedded into ALG-POL/SF gel to fabricate two kinds of BMP-7-loaded composite gels that were formulated with the exact same compositions as that respectively assigned to GB-2 and GB-3 gels (see Table 1), and major parameters for these gels are listed in Table 2.

**Figure 7:**
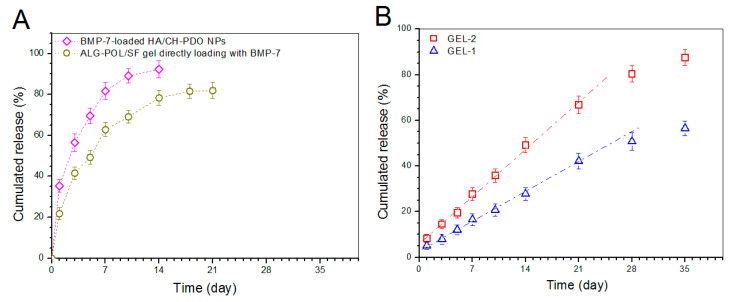
BMP-7 release profiles. (**A**) BMP-7-loaded HA/CH-PDO NPs and ALG-POL/SF gel directly loading with BMP-7; and (**B**) Gels embedded with BMP-7-loaded HA/CH-PDO NPs (see Table 2).

To make comparisons, BMP-7-loaded HA/CH-PDO NPs (total BMP-7 amount: 529.6 ng) and a kind of ALG-POL/SF gel directly loading with BMP-7 (total BMP-7 amount: 534.2 ng) were tested to determine their release profiles, and relevant data are depicted in Figure 7A. It can be seen that around 35% of the incorporated BMP-7 was released from NPs on the first day, and the cumulative BMP-7 reached about 60% for NPs after 3-day release. In cases of the gel directly loading with BMP-7, the initial burst release was reduced in comparison to NPs and its release pattern reached a plateau region after 2-week release. The results in Figure 7A reveal that BMP-7-loaded HA/CH-PDO NPs or the gel directly loading with BMP-7 are not suitable for administering the release of BMP-7 in view of their severe initial burst release characteristics and fast release rates.

Release patterns for gels embedded with BMP-7-loaded HA/CH-PDO NPs are illustrated in Figure 7B. The BMP-7 amount released from GEL-2 gel was measured to be around 8% in the first day, and BMP-7 was then released in an approximately linear way for about 3 weeks. GEL-1 gel behaved in a similar manner, namely, around 5% of BMP-7 was released in the first day, and thereafter, the BMP-7 release from GEL-1 gel followed a nearly linear trend for around 4 weeks at a markedly reduced release rate compared to that from GEL-2 gel. The BMP-7 release profiles clearly confirm that the presently constructed composite gels are able to control the BMP-7 release at varied rates in approximately linear manners for at least 3 weeks.

In many cases, hydrogels built with natural polymers have very limited ability to administer the controlled and sustained release of drugs or biomolecules [24,42]. By embedding BMP-7-loaded HA/CH-PDO NPs into ALG-POL/SF gel, the resulting composite gels show ability to administer the BMP-7 release in regulatory manners, as evidenced in Figure 7B. So achieved BMP-7 administration can be ascribed to the joint contribution of ALG-POL/SF gel and HA/CH-PDO NPs. Based on the compositions formulated for GEL-1 and GEL-2 gels, it can be envisioned that BMP-7 molecules need to diffuse out of HA/CH-PDO NPs first, and then to transport through ALG-POL/SF gel matrix to reach the release medium. During this procedure, BMP-7 molecules will face the resistance arisen from both HA/CH-PDO NPs and ALG-POL/SF gel matrix, which will inevitably slow down the BMP-7 release. It is worth mentioning that many preliminary experiments had been performed in this study to optimize parameters respectively matching with ALG-POL/SF gel and HA/CH-PDO NPs, which finally results in approximately linear BMP-7 release. The difference in release rates between GEL-1 and GEL-2 gels should be majorly attributed to their different initial BMP-7 loads. In general, a higher BMP-7 load in a gel would establish a steeper BMP-7 concentration gradient inside the gel during the release period, which would facilitate BMP-7 to faster diffuse through the gel matrix and to get into the media more rapidly when compared to the gel containing a low BMP-7 load.

### 3.4. Gene Expression Analyses

A variety of cell types have been investigated for their potential in cartilage repair. The typically employed seed cells include chondrocytes and stem cells derived from mesenchymal tissues [6,43]. Unlike cartilage tissue, the synovium has a high regenerative capacity, and it is easy to be obtained arthroscopically with minimal invasiveness. Moreover, a small amount of synovium tissue is sufficient to successfully isolate SMSCs [44]. In addition, studies symbolize that SMSCs are more prone to undergo chondrogenic differentiation in comparison to several other types of stem cells derived from mesenchymal tissues [6,7,8,9,10]. It is known that SMSCs express a number of surface markers [45,46]. Under current circumstances, selection of positive and negative markers for discriminating SMSCs was based on the minimal surface markers proposed by the International Society for Cellular Therapy [47]. Phenotypic analysis was conducted to detect the expression of several markers and results are elucidated in Figure S5. Flow cytometric detection demonstrated that the population of cells was negative for CD11b (MAC-1), CD45 (leukocyte common antigen), and positive for CD90 (Thy1.1), which validates their right phenotype in view of their similarity to that previously described for rat SMSCs [9].

**Figure 8:**
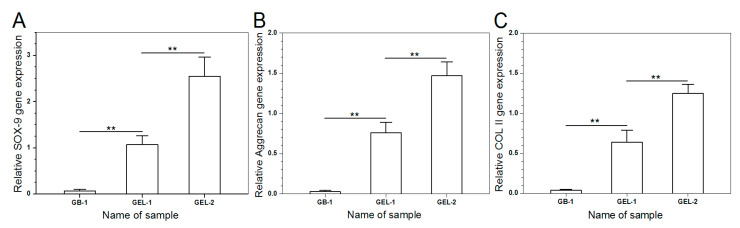
Chondrogenic gene expression of SOX-9 (**A**), aggrecan (**B**) and COL II (**C**) (**, *p* < 0.01).

Gene expression of chondrogenesis-relevant markers such as COL II (cartilage-specified differentiation), aggrecan (cartilage-related proteoglycan), and SOX-9 (early chondrogenic transcription factor) for 14-day cultured SMSCs were analyzed by semi-quantitative RT-PCR, and results are explicated in Figure 8. The expression levels of SOX-9, aggrecan and COL II were extremely low in GB-1 gel group, representing that SMSCs seeded in GB-1 gel are very hardly to spontaneously differentiate toward chondrogenesis under present culture conditions. On the other hand, SMSCs seeded in GEL-1 and GEL-2 gels clearly express SOX-9, aggrecan and COL II genes, and expression levels for GEL-1 and GEL-2 gels were much greater than that for GB-1 gel. In addition, significant differences in expression levels for three genes were registered between GEL-1 and GEL-2 gels, which can be assigned to the difference in the initial BMP-7 content for each gel and the different cumulative BMP-7 releases (see Table 2 and Figure 7B). Results in Figure 8 suggest that BMP-7 is able to induce the chondrogenic differentiation of SMSCs.

### 3.5. Proliferation of SMSCs

**Figure 9:**
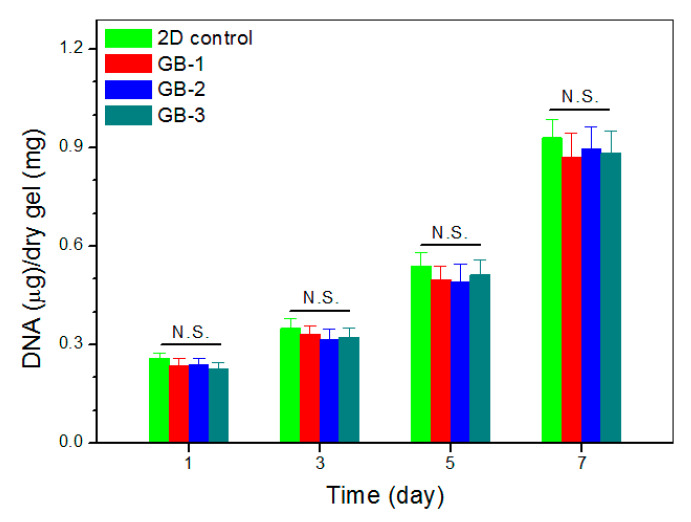
DNA content detected from cell-seeded gels during varied culture periods.

An important issue for the presently developed dual network gels is whether they are able to generate a suitable three-dimensional environment for supporting the growth of SMSCs. Blank ALG-POL/SF (see Table 1 for their composition) were thus seeded with SMSCs and cultured in vitro to determine cell proliferation, and relevant data are graphed in Figure 9. The bar-graph indicates that growth of cells was seen to roughly experience two phases: fewer cells grew from day 1 to day 3; and cells grew fast from day 5 to day 7. The slow cell growth in the first phase can be ascribed to the attachment of cells with population recovery; and the second phase confirms the occurrence of cell proliferation due to the growing DNA amount. Besides these, Figure 9 also shows that there were no significant differences in detected DNA amount among these gels, suggesting that they have similar abilities to support the proliferation of SMSCs even though they have some differences in their composition.

### 3.6. Deposition of Matrix Components

**Figure 10:**
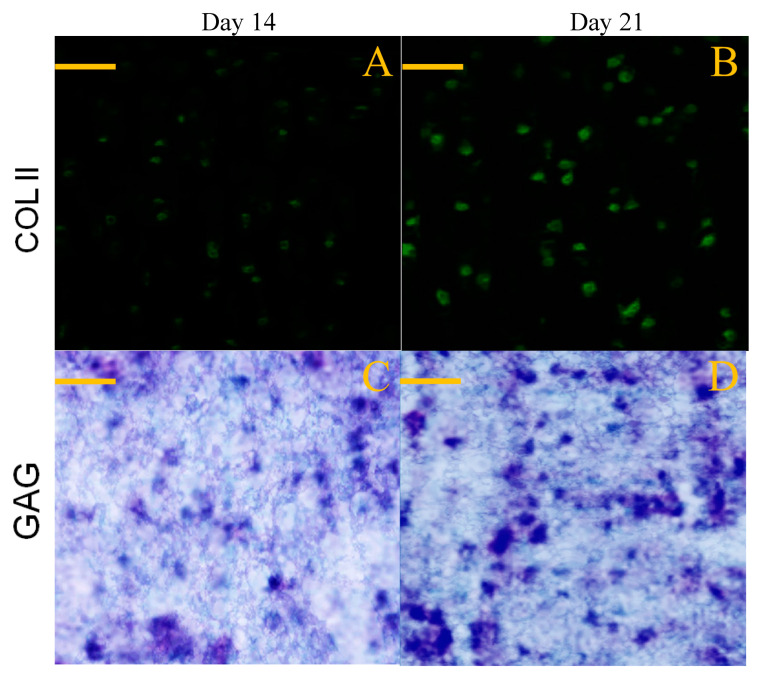
Immunofluorescence staining (**A**,**B**) for COL II and toluidine blue staining (**C**,**D**) for GAG (scale bar: 100 μm).

Chondrogenesis is known to associate with synthesis of several marker substances, typically including COL II, GAG and aggrecan molecules, which help in constructing the cartilaginous ECM [48]. GEL-2 gel seeded with SMSCs was cultured for varied periods up to 21 days, and used for examining the deposition of COL II and GAG components in consideration of its higher BMP-7 load compared to that for GEL-1 gel. Sections cut from the cell-seeded gels were stained and representative micrographs are represented in Figure 10. Images matching with COL II immunostaining showed significant COL II deposition in the gel, and toluidine blue staining confirmed the synthesis of GAG in the gel.

**Figure 11:**
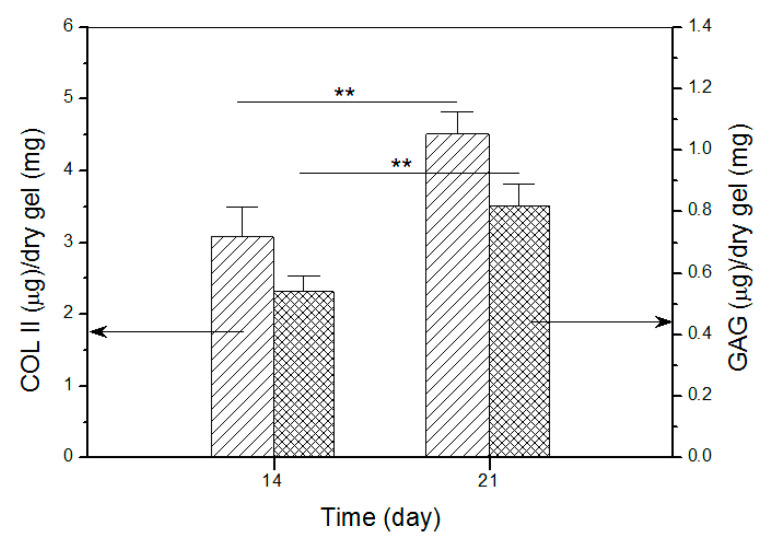
Detected amounts of COL II and GAG in cell-seeded gels (**, *p* < 0.01).

Images in Figure 10 roughly exhibited that fluorescence intensity for COL II and the stained blue area for GAG somewhat increased when incubation time was compared between 14 and 21 days. To make quantitative comparisons, amounts of COL II and GAG in cell-seeded gels were measured and relevant data are plotted in Figure 11. The bar-graphs in Figure 11 show that the production of both COL II and GAG significantly increased as culture time advanced from day 14 to day 21. The results delineated in Figure 10 and Figure 11 demonstrate that SMSCs seeded in GEL-2 gel already differentiate towards chondrogenesis.

Nowadays, several kinds of natural polymers, such as collagen, gelatin, silk fibroin, chitosan, alginate and hyaluronate, have been commonly used in the form of hydrogels for cartilage tissue engineering since they either contain the components existing in ECM or have certain similarities in structure and function to the components in ECM; and additionally, these polymers could be gelable under physiological conditions, enabling them to be advantageous over many kinds of gelable synthetic polymers [49,50]. ECM has important roles in regulating the development, function and homeostasis of all eukaryotic cells. In addition to providing physical support for cells, the ECM actively participates in the establishment, separation and maintenance of differentiated tissues and organs by regulating the abundance of growth factors and receptors, the level of hydration and the pH of the local environment [51]. In the case of cartilage repair, the hydrogels used to temporarily function as cartilage ECM have specifically to be mechanically strong, elastic and tough because chondrocytes in articular cartilage reside in a quite dynamic mechanical microenvironment and they need to endure repeated mechanical stimulation [51,52]. Despite the wide applicability, one of concerns associated with natural polymer hydrogels is correlated to their mechanical performance, including strength, elasticity and toughness because among them, there are still few gels concurrently having high strength, good elasticity and sufficient toughness [18,39,40,50]. In this study, the devised ALG-POL/SF gels are demonstrated to be mechanically strong, elastic and tough, signifying that these gels meet the basic mechanical requirements in cartilage repair. The BMP-7 incorporated inside the optimal gels can be controllably administered, and in particular, it is able to effectively induce the chondrogenic differentiation of seeded SMSCs, making the designed gel system specifically competent for use in cartilage tissue engineering.

Further mechanical investigations such as compression and tension measurements in incessant and long-cycle mode for these composite gels, the degradation of the gels and in vivo assessments for their potential in cartilage repair are currently in progress, and relevant results will be provided in a separate report.

## 4. Conclusions

A new type of dual network hydrogel composed of SF and ALG-POL was successfully fabricated by taking advantage of physical gelation stemmed from the ALG-POL component and covalently cross-linked gelation contributed by the SF component. The formulated ALG-POL/SF gel and its composites were found to be thermosensitive with their respective sol-gel transition features near physiological pH and temperature. The ALG-POL/SF gel and its composites were confirmed to be strong, elastic and tough, and the incorporation of HA/CH-PDO nanoparticles notably enhanced the strength but had no significant effect on the elasticity and toughness of the composite gels. The composite gels built through incorporation of BMP-7-loaded HA/CH-PDO nanoparticles showed abilities to administer BMP-7 release at different rates in approximately linear manners for a few weeks. The optimal BMP-7-loaded composite gel was able to induce the seeded SMSCs to differentiate toward chondrogenesis, and also, to support the synthesis of cartilaginous matrix components. Results suggest that this new type of dual network composite hydrogel has promising potency in cartilage repair.

## Figures and Tables

**Table 1:** Parameters for hydrogels without loading BMP-7.

Sample Name	SF (wt%)	ALG-POL (wt%)	Blank HA/CH-POD NPs (wt%) ^a^	H_2_O_2_ (µL) ^b^	HRP (µL) ^c^	pH	Gelation Time at 37 °C (min) ^d^
GB-1	7.0	5.0	−	10	10	7.14 ± 0.06	11.5 ± 0.57
GB-2	7.0	5.0	1.0	10	10	7.09 ± 0.08	10.25 ± 0.95
GB-3	7.0	5.0	2.0	10	10	7.12 ± 0.07	9.25 ± 0.5

^a^ See Section 3.1 for their parameters; ^b^ concentration of H_2_O_2_: 500 mmol/L; ^c^ concentration of horseradish peroxidase (HRP): 1000 U/mL; ^d^ gelation time was assessed by inverting the vial every 1 min.

**Table 2:** Parameters for hydrogels loading with BMP-7.

Sample Name	SF (wt%)	ALG-POL (wt%)	BMP-7 Loaded HA/CH-POD NPs (wt%) ^a^	H_2_O_2_ (µL) ^b^	HRP (µL) ^c^	BMP-7 Content in Gel (μg/mL)
GEL-1	7.0	5.0	1.0	10	10	1.07 ± 0.11
GEL-2	7.0	5.0	2.0	10	10	2.18 ± 0.14

^a^ BMP-7 load in HA/CH-POD NPs was regulated by changing the BMP-7 feed amount. ^b^ and ^c^ See Table 1 for their concentrations.

## Supplementary Materials

The following are available online at https://www.mdpi.com/1999-4923/12/7/613/s1, Figure S1: FTIR spectra for POL, ALG and ALG-POL, Figure S2: Strain sweep curves (A, B and C) for ALG-POL gels having varied concentrations and their average yielding strains (D) (N.S., no significance), Figure S3: Frequency-dependent curves of G′ and G′′ for ALG-POL/SF composite gels without BMP-7 loading (see Table 1 for their parameters), Figure S4: Shear-rate dependent functions of viscosity (23 °C) for ALG-POL/SF composite gels without BMP-7 loading (see Table 1 for their parameters), Figure S5: FCM histograms of fluorescence intensity for cells, Table S1: Primer sequences used for RT-PCR analysis.
