# Supplementary material for: Dual Network Hydrogels Incorporated with Bone Morphogenic Protein-7-Loaded Hyaluronic Acid Complex Nanoparticles for Inducing Chondrogenic Differentiation of Synovium-Derived Mesenchymal Stem Cells
Source: Pharmaceutics. 2020 Jun 30;12(7):613. doi: 10.3390/pharmaceutics12070613 (PMC7407334; doi:10.3390/pharmaceutics12070613)
Supplement: Supplementary file 1 [file pharmaceutics-12-00613-s001.pdf]

pharmaceutics

Supplementary Materials: Dual Network Hydrogels Incorporated with Bone Morphogenic Protein-7-Loaded Hyaluronic Acid Complex Nanoparticles for Inducing Chondrogenic Differentiation of Synovium-Derived Mesenchymal Stem Cells

Qing Min, Jiaoyan Liu, Yuchen Zhang, Bin Yang, Ying Wan and Jiliang Wu

1. Preparation of Silk Fibroin

SF was produced using Bombyx Mori cocoons according to the reported method [1]. Bombyx Mori cocoons were purchased from Anbo Pharmaceutical Ltd (Haozhou, China). Cocoons (3 g) were cut into small pieces, and degummed in 1200 mL of Na_2CO_3 aqueous solution (0.02 M) at 100 °C for 30 min. The collected silk fibroin fibers were washed repeatedly with deionized water, and then, air-dried in a fume hood for 2 days. The degummed silk fibroin fibers were further dissolved in an LiBr aqueous solution (9.3 M) at 60 °C for 4 h, and the resulting solution was dialyzed against deionized water for 2 days using membrane tubes (MWCO: 3500). Afterwards, the SF solution was centrifuged twice at 9000rpm each for 20min to remove insoluble granules, concentrated to a concentration of around 10% by dipping the solution-loaded membrane tubes into a 50% PEG20000 solution.

2. Synthesis of Monoamine-Terminated Poloxamer (MATP)

The intermediate, namely, monoamine-terminated poloxamer (MATP), was first synthesized following reported methods [2,3] In a typical synthesis procedure, 5g of poloxamer and 0.2g of 4-nitrophenyl chloroformate were dissolved in 50 mL of CH_2Cl_2 in the presence of triethylamine (0.15 mL). This solution was allowed to react at room temperature for 4 h. After that, the mixture was subjected to rotary evaporation at 40 °C for removal of solvent, washed with petroleum ether, and vacuum-dried to obtain the white intermediate. To a mixed solution composed of ethylene diamine (1 mL) and methylene chloride (50 mL), the retrieved intermediate (5g) was added, and reaction was conducted at room temperature for 12 h with stirring. Afterwards, the mixture was extracted three times with petroleum ether, dialyzed against double-distilled water (ddH_2O) using a membrane tube (MWCF: 3500) for 3 days, and lyophilized to achieve MAPT.

3. Synthesis of Alginate-Poloxamer Copolymers

Alginate-poloxamer (ALG-POL) copolymers were synthesized by coupling MATP onto alginate via EDC/NHS chemistry. In brief, alginate and MATP (4.5 g) were dissolved in ddH_2O (50 mL) at a weight ratio of alginate to MATP at 1:30. To this mixture, EDC (0.46 g) and NHS (1.38 g) were introduced and the reaction was carried out at ambient temperature for 24 h with stirring. After reaction, the mixture was dialyzed against deionized water using a membrane tube (MWCF: 12-15k) for 3 days, and freeze-dried to obtain the ALG-POL copolymer.

4. FTIR Analysis of ALG-POL

The preliminary experimental results showed that the composition of ALG-POL exerted significant effects on its thermal transition temperature, degradation resistance and the strength of resulting dual network ALG-POL/SF gels. Accordingly, the compositional proportion of ALG and POL components in ALG-POL copolymer was optimized via orthogonal design method. The POL

content in the optimal ALG-POL copolymer was selected as about 66 wt% by controlling the alginate/MATP ratio at 1:30, which was determined by an elemental analyzer (Vario EL III, Elementar). Fourier transform infrared (FTIR) analyses of POL, ALG and ALG-POL were performed on a spectrometer (Vertex 70, Bruker, Germany), and their FTIR spectra are presented in Figure S1.

The spectrum of POL is characterized by three typical bands at 2891 (C-H stretch aliphatic), 1345 (in-plane O-H bend) and 1112 cm^{-1} (C-O stretching). The ALG spectrum shows specific absorbance bands of its COOH stretching at 1610 cm^{-1} and C-O-C stretching at 1305 cm^{-1} [4], respectively. In the spectrum for ALG-POL, the carbonyl absorption band for carboxylate sodium salt originally showing in the ALG spectrum disappeared while a new characteristic amide I band appeared at around 1637 cm^{-1}, suggesting that amide bonds have formed between ALG and POL [5]. FTIR results demonstrate that the ALG-POL copolymer has been successfully synthesized.

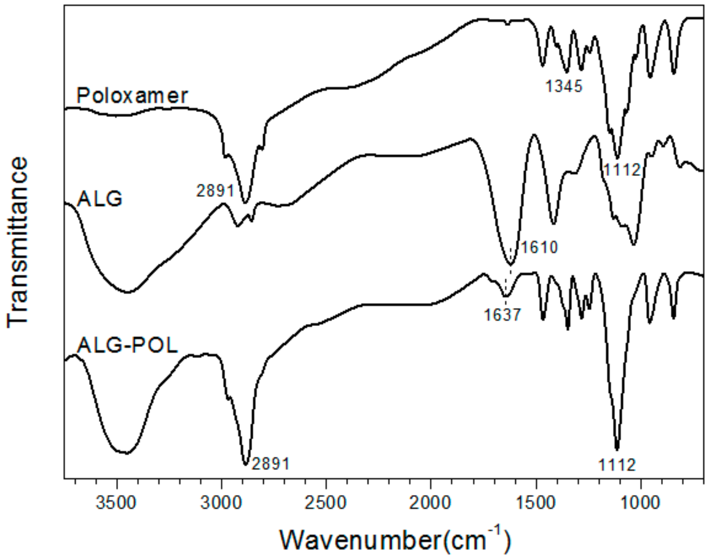

Figure S1. FTIR spectra for POL, ALG and ALG-POL.

Table S1. Primer sequences used for RT-PCR analysis.

Gene Name	Forward Primer (5′-3′)	Reverse Primer (5′-3′)
SOX-9	AGCGAACGCACATCAAGAC	GCTGTAGTGTGGGAGGTTGAA
Aggrecan	TGCATTCCACGAAGCTAACCTT	GACGCCTCGCCTTCTTGAA
COL II	CCATGTGACCATGAGGAAAT	CGATAACAGTCTTGCCCCACTT
GAPDH	AGAAAAACCTGCCAAATATGATGAC	TGGGTGTCGCTGTTGAAGTC

A

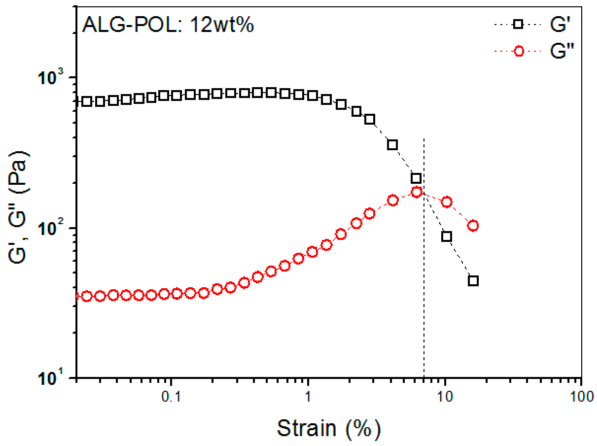
ALG-POL: 12wt%
G'
G"
G', G" (Pa)
Strain (%)

B

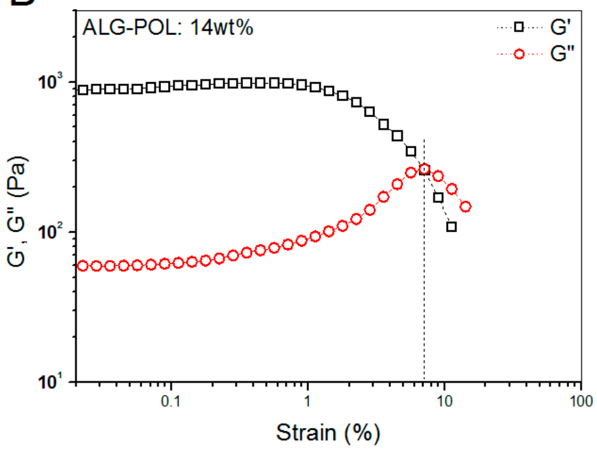
ALG-POL: 14wt%
G'
G"
G', G" (Pa)
Strain (%)

C

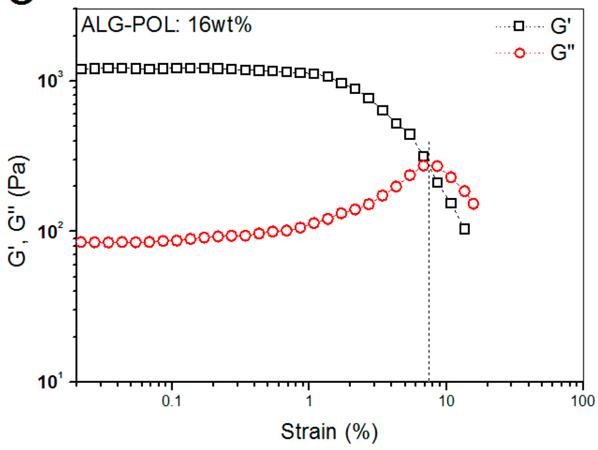
ALG-POL: 16wt%
G'
G"
G', G" (Pa)
Strain (%)

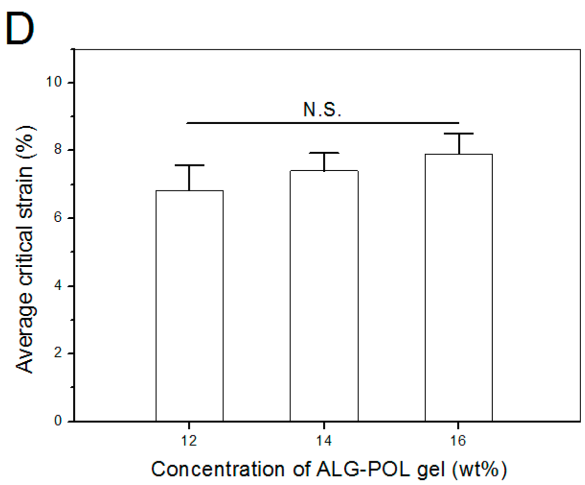

Figure S2. Strain sweep curves (**A**, **B** and **C**) for ALG-POL gels having varied concentrations and their average yielding strains (**D**) (N.S., no significance).

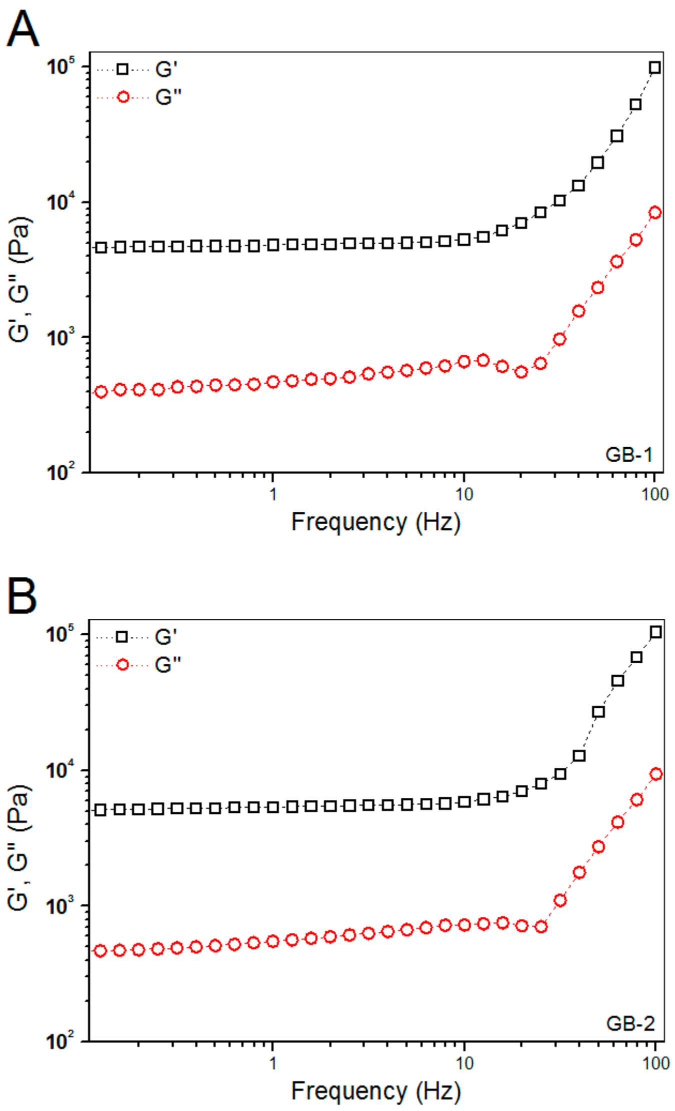

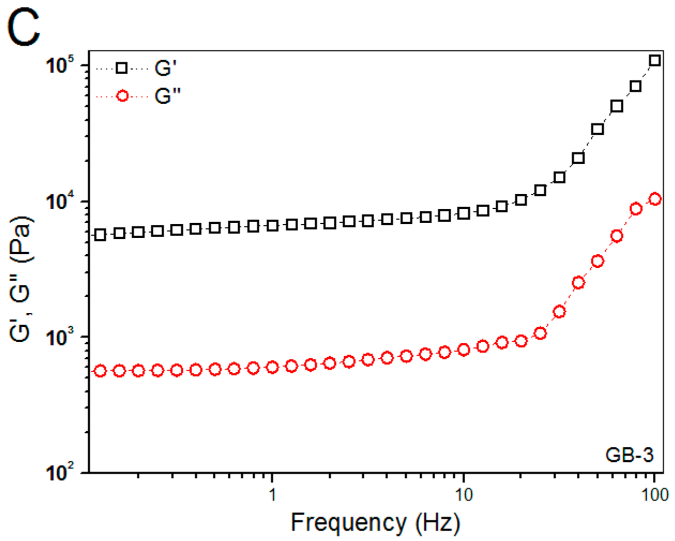

Figure S3. Frequency-dependent curves of G′ and G″ for ALG-POL/SF composite gels without BMP-7 loading (see Table 1 for their parameters).

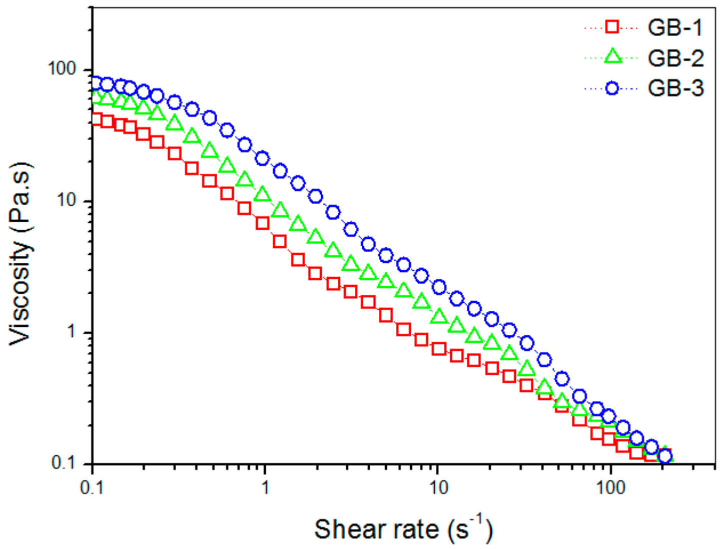

Figure S4. Shear-rate dependent functions of viscosity (23 °C) for ALG-POL/SF composite gels without BMP-7 loading (see Table 1 for their parameters).

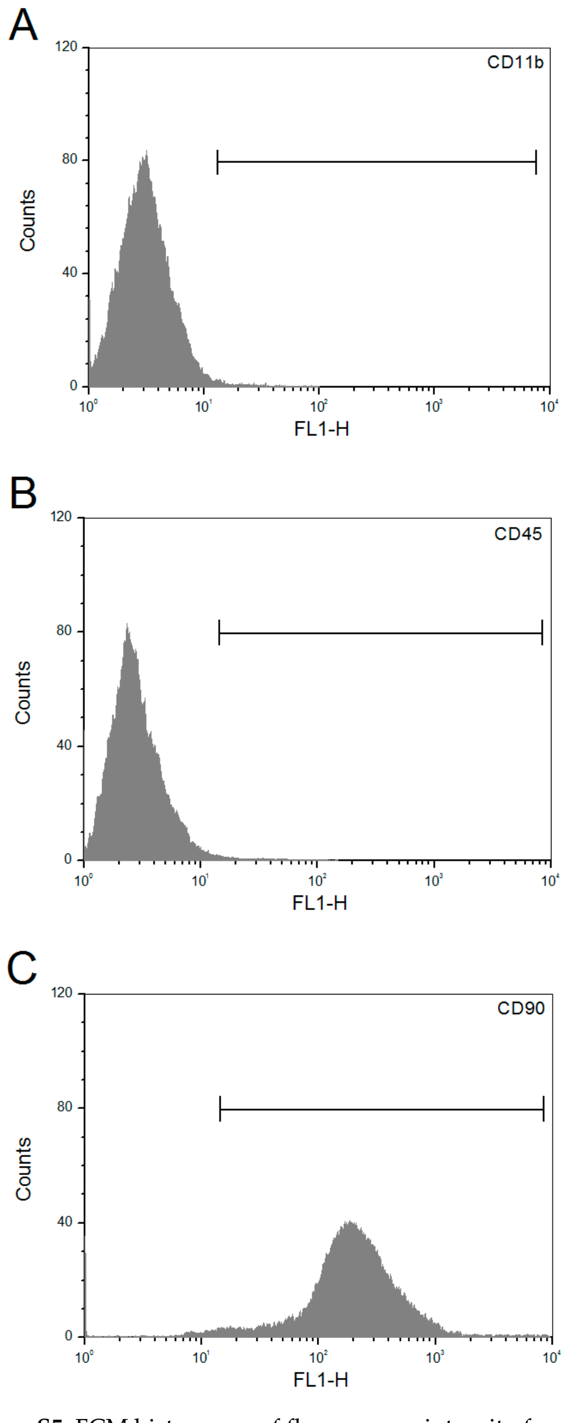

Figure S5. FCM histograms of fluorescence intensity for cells.

References

1. Hopkins, A.M.; Laporte, L.D.; Tortelli, F.; Spedden, E.; Staii, C.; Atherton, T.J.; Hubbell, J.A.; Kaplan, D.L. Silk hydrogels as soft substrates for neural tissue engineering. *Adv. Funct. Mater.* **2013**, *23*, 5140–5149.
2. Cho, K.Y.; Chung, T.W.; Kim, B.C.; Kim, M.K.; Lee, J.H.; Wee, W.R.; Cho, C.S. Release of ciprofloxacin from poloxamer-graft-hyaluronic acid hydrogels in vitro. *Int. J. Pharm.* **2003**, *260*, 83–91.
3. Hsu, S.H.; Leu, Y.L.; Hu, J.W.; Fang, J.Y. Physicochemical characterization and drug release of thermosensitive hydrogels composed of a hyaluronic acid/pluronic F127 graft. *Chem. Pharm. Bull.* **2009**, *57*, 453–458.
4. Fang, J.Y.; Hsu, S.H.; Leu Y.L.; Hu, J.W. Delivery of cisplatin from pluronic co-polymer systems: Liposome inclusion and alginate coupling. *J. Biomater. Sci, Polym. Ed.* **2009**, *20*, 1031–1047.
5. Chen, C.C.; Fang, C.L.; Al-Suwayeh, S.A.; Leu, Y.L., Fang, J.Y. Transdermal delivery of selegiline from alginate-pluronic composite thermogels. *Int. J. Pharm.* **2011**, *415*, 119–128.
